# Development of Safe and Non-Self-Immunogenic Mucosal Adjuvant by Recombinant Fusion of Cholera Toxin A1 Subunit with Protein Transduction Domain

**DOI:** 10.1155/2018/9830701

**Published:** 2018-03-07

**Authors:** Byoung-Shik Shim, In Su Cheon, Eugene Lee, Sung-Moo Park, Youngjoo Choi, Dae-Im Jung, Eunji Yang, Jung-ah Choi, June Young Chun, Jae-Ouk Kim, Cheol-Heui Yun, Cecil Czerkinsky, Man Ki Song

**Affiliations:** ^1^Laboratory Science Division, International Vaccine Institute, Seoul 08826, Republic of Korea; ^2^Department of Agricultural Biotechnology and Research Institute for Agriculture and Life Sciences, Seoul National University, Seoul 08826, Republic of Korea; ^3^Department of Internal Medicine, Seoul National University College of Medicine, Seoul, Republic of Korea; ^4^Institut de Pharmacologie Moleculaire et Cellulaire, CNRS-INSERM-University of Nice-Sophia Antipolis, Valbonne, France

## Abstract

Potential use of cholera toxin (CT) as a mucosal vaccine adjuvant has been documented in a variety of animal models. However, native CT is highly toxic to be used as a mucosal adjuvant in humans. Here, we demonstrate a new approach to generate a mucosal adjuvant by replacing the B subunit of CT with HIV-1 Tat protein transduction domain (PTD), which efficiently delivers fusion proteins into the cell cytoplasm by unspecific binding to cell surface. We compared the adjuvanticity and toxicity of Tat PTD-CTA1-Tat PTD (TCTA1T) with those of CT. Our results indicate that intranasal (i.n.) delivery of ovalbumin (OVA) with TCTA1T significantly augments the OVA-specific systemic and mucosal antibody responses to levels comparable to those seen with CT adjuvant. Moreover, *in vivo* cytotoxic T lymphocyte activity elicited by TCTA1T was significantly higher than that elicited by a mutant TCTA1T (TmCTA1T) lacking ADP-ribosyltransferase function. In addition, coadministration of influenza M2 protein with TCTA1T conferred near complete protection against lethal influenza virus challenge. Importantly, TCTA1T, in contrast to CT, did not induce serum IgG antibody responses to itself and was shown to be nontoxic. These results suggest that TCTA1T may be a safe and effective adjuvant when given by mucosal routes.

## 1. Introduction

Mucosal surfaces function as a barrier between the host interior and the external environment [1]. Since most pathogens invade the body through the mucosal epithelium [2], mucosal immunity serves a crucial role as the first line of defense against various infections [3]. A general consensus is that parenteral administration of vaccines generates a systemic immune response against the vaccine antigens (Ags), whereas mucosal vaccination can efficiently elicit both systemic and mucosal immune responses, including secretory IgA (sIgA) antibodies which are strongly associated with mucosal host defense [2, 4]. In addition, mucosal immunization possesses a number of advantages, including ease of administration and reduced risks of infection by contaminated injection devices. Nonetheless, only few mucosal vaccines have been licensed for human use and no mucosal adjuvant has yet been approved [2, 5]. Despite their obvious advantages, the evident challenge faced by many candidate mucosal vaccines has been their inability to elicit adequate immune responses when administered via mucosal routes [6, 7]. Such a challenge indicates that these candidate vaccines may require the use of proper adjuvant to efficiently generate immune responses that can offer protection against invading pathogens.

It is well known from animal studies that cholera toxin (CT) secreted by *Vibrio cholerae* and heat-labile enterotoxin (LT) from *Escherichia coli* (*E. coli*) retain potent mucosal adjuvanticity which helps to elicit both mucosal and systemic antibody (Ab) responses against variety of Ags [8–12]. Both CT and LT are composed of enzymatically active A subunit which possesses ADP-ribosylating activity and pentameric B subunits which possesses monosialoganglioside (GM1) receptor-binding site. The A1 subunit (CTA1) of CT is internalized following binding of B subunits of CT to GM1 on the surface of intestinal epithelial cells [13]. Then, the internalized CTA1 induced ADP-ribosylation of the *α* subunit of the GTP binding regulatory protein Gs, resulting in the increased levels of cellular cyclic AMP (cAMP) which, in turn, cause secretion of chloride ions and water into the small intestine [14], the hallmark of cholera diarrhea in humans [14, 15]. Because native CT and LT are not suitable as mucosal adjuvants in humans [16], a number of toxin-derived mutants, including LTR192G, LTK63, and CTA1-DD, have been engineered so as to retain adjuvanticity without toxicity [1, 3, 17, 18]. Of these, LTR192G and LTK63 have already been evaluated in human clinical trials [19, 20].

It has been suggested that one of the important requirements for an ideal adjuvant is nonimmunogenicity to itself. This is due to potential development of the adjuvant-specific immunity capable of negating the immune-enhancing functionality of the adjuvant. Although adjuvant functionality of toxin-derived mutants, notably LTK63 and CTA1-DD, was not affected by preexisting antibodies to the adjuvants themselves [21, 22], a previous study reported that preexisting immunity to CTB can inhibit antibody responses to a coadministered Ag [23, 24].

It has been known that protein delivery by so-called “protein transduction domain” (PTD) occurs in a rapid, concentration-dependent manner that appears to be independent of cell membrane receptors and cellular transporters [25, 26]. Thereafter, PTD has been considered an ideal delivery vehicle that enables efficient transport of PTD-fusion proteins into living cells [26, 27]. Previous studies have shown that the PTD comprising nine amino acids (residues 49–57) from HIV-1 Tat protein is sufficient for the delivery of PTD-GFP fusion protein into cells [28] and that GFP fused with PTD at both termini displays increased translocation activity as compared to GFP fused with PTD at either N- or C-terminus alone [29].

In the above, considerations have prompted us to evaluate the adjuvant activity and potential toxicity of a novel mucosal adjuvant engineered by fusion of the HIV-1 Tat PTD to both termini of CTA1, which will be referred thereafter to as TCTA1T. In addition, we also examined whether TCTA1T is self-immunogenic, that is, can induce antibody responses to itself.

## 2. Materials and Methods

### 2.1. Construction of Plasmids Expressing TCTA1T and TmCTA1T Proteins

**Figure 1:**
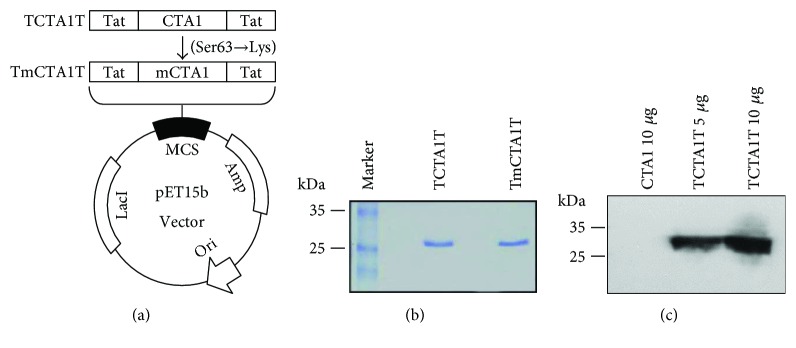
Construction, purification, and transduction of the recombinant proteins. (a) Construction of plasmids expressing the proteins based on fusion of HIV-1 Tat PTD at termini of CTA1 or mCTA1. TmCTA1T was generated from TCTA1T by site-directed mutagenesis. (b) The fusion proteins expressed in *E. coli* were purified by His-tag affinity chromatography and separated by 12.5% SDS-PAGE. (c) The purified proteins were added to HeLa cells, and the presence of transduced proteins into the cells was detected by Western blot analysis.

Plasmid (pET15b-Tat-GFP-Tat) expressing GFP protein with HIV-1 Tat PTD (amino acids 49–57) at both N-terminus and C-terminus was provided by Dr. Soo Young Choi at Hallym University, Korea. The gene corresponding to CTA1 subunit (amino acids 1–194) was amplified with a forward primer (5′-GGGCCCCTCGAGAATGATGATAAGTTATATCGG-3′) and a reverse primer (5′-CCCGGGGGATCCCGATGATCTTGGAGCATTCCC-3′) by polymerase chain reaction (PCR). *Vibrio cholerae* N16961 strain (AE003852) was used as a template for CTA1 subunit. The PCR product was digested with Xho I and BamH I and then ligated to the pET15b-Tat-GFP-Tat plasmid which was linearized with the same enzymes, resulting in the recombinant plasmid pET15b-TCTA1T. Plasmid pET15b-TmCTA1T, which has a point mutation (Ser63→Lys) at ADP-ribosyltransferase enzymatic active site of CTA1, was generated by site-directed mutagenesis with 5′-TATGTTTCCACCAAGATTAGTTTGAGA-3′ and 5′-TCTCAAACTAATCTTGGTGGAAACATA-3′ primers using Pyrobest DNA polymerase (Takara, Japan). The pET15b-TCTA1T plasmid was used as a template for TmCTA1T (Figure 1(a)), and the DNA sequences were confirmed at Macrogen (Seoul, Korea).

### 2.2. Expression and Purification of TCTA1T and TmCTA1T Proteins

*E. coli* BL21 (DE3) strain (Novagen, Germany) was transformed with the pET15b-TCTA1T or pET15b-TmCTA1T and was grown overnight at 37°C in Luria-Bertani (LB) medium supplemented with 100 *μ*g/ml of ampicillin. The overnight culture was transferred into fresh LB medium and cultured at 37°C while shaking at 180 rpm until OD_600_ of 0.6~0.8. Each protein expression was induced by addition of isopropyl *β*-D-thiogalactopyranoside (IPTG) to a final concentration at 0.5 M for 4 hrs, and the cells were harvested by centrifugation at 6000 rpm for 10 min. The cell pellets were suspended in binding buffer (20 mM Tris, 0.5 M NaCl, 10% glycerol, pH 7.9) and disrupted by sonication on ice. Then, the soluble and insoluble fractions were separated by centrifugation for 30 min at 18,000 rpm. The insoluble fraction was dissolved in binding buffer containing 6 M urea. After centrifugation for 30 min at 18,000 rpm, the supernatant was applied to a Talon metal affinity column (Clontech, Palo Alto, CA). The column was washed with binding buffer, followed by wash buffer (20 mM Tris, 0.5 M NaCl, and 20 mM imidazole, pH 7.9) without urea. Then, the proteins were eluted with elution buffer (20 mM Tris, 0.5 M NaCl, 0.3 M imidazole, 10% glycerol, pH 7.9). The purified proteins were electrophoresed on 12.5% SDS-PAGE, and the protein bands were visualized by staining with Coomassie Brilliant Blue. The protein concentration was determined by Bradford protein assay kit (Bio-Rad, Richmond, CA). The purified proteins were aliquoted and stored at −80°C until used.

### 2.3. Western Blot Analysis

The purified proteins were separated by 12.5% SDS-PAGE. After electrophoretic transfer to nitrocellulose membrane (Schleicher & Schuell, Germany) by using a semidry transblot apparatus (Bio-Rad), the membrane was blocked with Tris-buffered saline (TBS) containing 5% skim milk and incubated with goat anti-CT (Abcam, Cambridge, MA) at a 1 : 1000 dilution in TBST (TBS and 0.05% Tween 20) containing 5% skim milk. After washing with TBST, the membrane was probed with mouse-anti-goat IgG conjugated to horseradish peroxidase (Santa Cruz Biotechnology, Santa Cruz, CA) for 1 hr and the band was visualized after the reaction with chromogenic substrate (ECL kit; Amersham Pharmacia Biotech Inc., Piscataway, NJ).

### 2.4. Transduction of TCTA1T into Cells

Analysis of the transduction of TCTA1T into cells was performed as previously described [28]. Briefly, HeLa cells were seeded at 5 × 10^5^ cells/well in 6-well plates. 24 hrs later, the cells were treated with 5 *μ*g CTA1 or 5 and 10 *μ*g TCTA1T and incubated in CO_2_ incubator for 2 hrs. The cells were washed with PBS three times and harvested by trypsinization. The cells were prepared for analysis by Western blot.

### 2.5. Immunization of Mice

Specific pathogen-free, female BALB/c mice aged 6 weeks were purchased from Orient Bio Inc. (Korea). All mice were maintained under specific pathogen-free conditions, and all studies were approved by Institutional Animal Care and Use Committee (IACUC) of International Vaccine Institute (2010-018). Five mice per group were anesthetized with ketamine and immunized three times at a 2-week interval by i.n. injection of 20 *μ*g of ovalbumin (OVA; Sigma, St. Louis, MO) alone, or mixed with 10 *μ*g of TCTA1T and TmCTA1T, or 2 *μ*g of CT (List Biological Laboratories Inc., Campbell, CA). To examine adjuvant effect depending on the dose of TCTA1T, mice were immunized three times at a 2-week interval by i.n. injection with 20 *μ*g of OVA alone, or mixed with 0.1, 1.0, 10, or 20 *μ*g of TCTA1T. The treated mice were monitored daily and euthanized according to ethical guidelines of the IACUC as per the approved protocol.

### 2.6. Sample Collection

Sera and mucosal samples were collected on day 13 or 14 after the last immunization. Blood samples were collected from the retro-orbital plexus and centrifuged for 10 min at 13000 rpm, and sera were taken. Saliva samples were obtained after inducing salivary gland secretion by intraperitoneal (i.p.) injection of pilocarpine (100 *μ*l of 1 mg/ml; Sigma) diluted in sterile PBS. For Bronchoalveolar lavage (BAL) samples, the mice were dissected to expose the trachea. IV catheter (BD Biosciences, San Jose, CA) was inserted into a small nick of the trachea. BAL samples were collected by repeated flushing and aspiration with 500 *μ*l of PBS into the lungs. Nasal washes were collected by flushing with 50 *μ*l of PBS for two times through the nasal cavity. Lung tissues were cut in small pieces and subjected to freeze-thaw cycles twice. The tissues were centrifuged at 13000 rpm at 4°C for 10 min, and supernatant was collected to test for Ag-specific Ab responses. The samples were stored at −80°C until used.

### 2.7. Elisa

OVA-specific Ab titers were determined by ELISA. 96-well ELISA plates (Nunc, Roskilde, Denmark) were precoated with 100 *μ*l of OVA protein (10 *μ*g/ml) in 50 mM sodium bicarbonate buffer (pH 9.6) overnight at 4°C. After blocking with PBS containing 5% skim milk for 1 hr at room temperature, 100 *μ*l of 2- or 3-fold serially diluted samples in blocking buffer were added to each well and incubated for 1 hr at 37°C, followed by addition of 1 : 3000 diluted horseradish peroxidase-conjugated goat anti-mouse IgG, IgG1, IgG2a, or IgA (Santa Cruz biotechnology). After incubation for 1 hr at room temperature, 100 *μ*l of peroxidase substrate tetramethylbenzidine (TMB) (Millipore, Bedford, MA) was added to each well. The reaction was stopped by addition of 0.5 N HCl. The absorbance at wavelength 450 nm was examined by using an ELISA reader (Molecular Devices, Sunnyvale, CA). The endpoint titer was determined by O.D. cutoff values of 0.2.

### 2.8. *In Vivo* Cytotoxic T Lymphocyte Assay

Splenocytes from C57BL/6 mice were split into two equal fractions. One fraction was labeled with 5 *μ*M CFSE (Invitrogen) for 5 min at room temperature and pulsed with 1 *μ*M OVA_257–264_ (SIINFEKL) peptide for 1 hr. The other fraction was labeled with 0.5 *μ*M CFSE without peptide pulse. Two fractions were mixed at a ratio of 1 : 1, and a total of 1.5 × 10^7^ cells were injected intravenously (i.v.) into C57BL/6 mice, which were previously immunized three times at a 2-week interval by i.n. injection with 100 *μ*g of OVA alone, or OVA plus 10 *μ*g of TCTA1T or TmCTA1T, or 2 *μ*g of CT. Single cells were prepared from the lung and spleen 24 hrs after the cell transfer. Specific killing activity was measured by FACSCalibur™ (BD Biosciences, San Jose, CA).

### 2.9. Virus Challenge

BALB/c mice (*n* = 6) were immunized with 10 *μ*g of influenza 3M2eC protein [30] with 10 *μ*g of TCTA1T and TmCTA1T or 2 *μ*g of CT as mucosal adjuvant by i.n. route on days 0 and 14. Control mice were immunized with either PBS or TCTA1T alone. The mice were challenged i.n. with 10 LD_50_ of A/PR/8 virus three weeks after the last immunization. The mice were monitored daily for body weight loss and survival after the viral challenge.

### 2.10. Toxicity Test

To assess the footpad edema [31], mice were anesthetized with ketamine by i.p. injection and injected with 10 *μ*g of TCTA1T and TmCTA1T, or 1 *μ*g of CT in 10 *μ*l of PBS into the hind paw. The thickness of the footpad was measured after 24 hrs.

To perform the intestinal loop test [32], mice were deprived of food for overnight, but not of water. Next day, the mice were anesthetized, the abdomen was opened, and 3 to 5 cm loop was ligated in the middle part of the small intestine. 2 *μ*g of CT and 10 *μ*g of TCTA1T and TmCTA1T in 100 *μ*l of PBS were injected into the loops. PBS was used as a control. The abdomen was closed, the loop was weighed, and its length was determined 6 hrs after the injection. Values were expressed as the weight per length ratio (mg/cm).

To test induction of cAMP accumulation, BHK21 cells (ATCC number CCL-10) were seeded at 1 × 10^6^ cells/well in a 6-well plate. 24 hrs later, the cells were washed with serum-free medium and incubated in the presence of 10 *μ*g of TCTA1T and TmCTA1T, or 1 *μ*g of CT for 3 hrs. Supernatant were obtained by centrifugation at 1000 ×g for 10 min. The concentration of cAMP in the supernatant was measured by cyclic AMP EIA kit (Cayman, Michigan, MA) according to the manufacturer's instructions.

### 2.11. Toxicity Test of TCTA1T in Lung Tissue

To further assess toxicity of TCTA1T adjuvant in lung tissue [33], the mice were injected i.n. with 10 and 50 *μ*g of TCTA1T, or 1, 5, and 10 *μ*g of CT. PBS was used as control. The mice were monitored daily for the body weight loss following the injection. Lung tissues were weighed on day 4 after the injection.

### 2.12. Self-Immunogenic Response to TCTA1T

To evaluate self-immunogenicity of TCTA1T, BALB/c mice were immunized i.n. or intradermally (i.d.) for three times at 2 weeks apart with 10 *μ*g of TCTA1T or 2 *μ*g of CT. The sera were harvested at 2 weeks after the last immunization. The 96-well ELISA plates were precoated with CTA1 (1 *μ*g/ml) or CT (2 *μ*g/ml) and CTA1- or CT-specific Ab titers were determined by ELISA as described above.

### 2.13. Statistical Analysis

Statistical tests were subjected to one-way analysis of variance (ANOVA) using SPSS software (SPSS Inc., Chicago, IL). A *P* value of less than 0.05 was considered significant.

## 3. Results

### 3.1. Construction and Purification of Recombinant TCTA1T and TmCTA1T Proteins

A previous study has demonstrated that dual attachment of PTD onto both termini of GFP enhances the cellular uptake compared to the single PTD attachment at either terminus [29]. Accordingly, we constructed our candidate adjuvant TCTA1T by fusing HIV-1 Tat PTD onto both N-terminus and C-terminus of CTA1 subunit, which consequently allowed bypassing of CTB-dependent cellular internalization of CTA1 (Figure 1(a)). Also, TmCTA1T, which has a point mutation (Ser63→Lys) at ADP-ribosyltransferase enzymatic active site within CTA1 [34], was constructed from TCTA1T backbone via site-directed mutagenesis and used as negative control (Figure 1(a)). Both proteins were expressed in *E. coli* BL21 (DE3) strain and purified by His-tag affinity chromatography. The purified proteins were then analyzed by SDS-PAGE which displayed distinct bands at the expected molecular weight of ~27 kDa (Figure 1(b)). Further, the presence of proteins with correct size in the purified samples was confirmed by Western blot analysis performed using anti-CTAl Ab (data not shown).

### 3.2. Translocation of Recombinant TCTA1T Protein into Cells

To investigate whether TCTA1T possesses the capacity to translocate into target cells, HeLa cells were treated with 5 or 10 *μ*g TCTA1T or 5 *μ*g wild-type CTA1 (lacking HIV-1 Tat PTD fusion) for 2 hrs. Following incubation, cells were washed thoroughly with PBS to eliminate any residual TCTA1T or wild-type CTA1 from the growth media before being harvested and lysed. Cell lysates were analyzed for the presence of TCTA1T by Western blot. As expected, the presence of TCTA1T was detected in the lysates of cells treated with 5 or 10 *μ*g TCTA1T in a dose-dependent manner (Figure 1(c)). Whereas, the CTA1 without Tat PTD domain was not detected (Figure 1(c)). This result indicates that TCTA1T is able to permeate across the target cell membrane independently of the CTB domains which functions as a ligand for GM1 ganglioside receptor.

### 3.3. TCTA1T Enhances OVA-Specific Systemic and Mucosal Ab Responses

**Figure 2:**
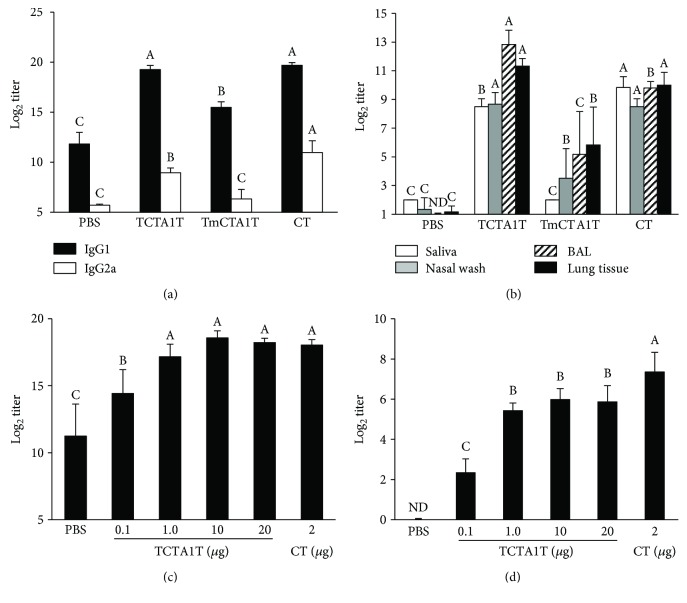
Intranasal TCTA1T enhances OVA-specific systemic and mucosal immune responses. (a and b) BALB/c mice were immunized i.n. with 20 *μ*g of OVA alone, plus 10 *μ*g of TCTA1T and TmCTA1T or 2 *μ*g of CT on days 0, 14, and 28. Samples were collected on day 13 or 14 after the last immunization. The levels of OVA-specific IgG1 and IgG2a in sera (a) and IgA in mucosal external secretions (b) were determined by ELISA. (c and d) BALB/c mice were immunized three times at 2-week intervals by i.n. injection with 20 *μ*g of OVA alone, or plus 0.1, 1.0, 10, and 20 *μ*g of TCTA1T, or 2 *μ*g of CT. The levels of OVA-specific IgG in sera (c) and IgA in saliva (d) were determined by ELISA. ND, not detectable. The results are expressed as mean ± SD for the group (*n* = 5 to 6). The data are representative of three separate experiments. The different letters within the same samples are significantly different (*P* < 0.05).

In order to examine the efficiency of TCTA1T in enhancing Ag-specific humoral immune response when used as a mucosal adjuvant, BALB/c mice were immunized three times i.n. with OVA alone, or OVA mixed with TCTA1T, TmCTA1T, or CT at 2-week intervals. Sera and mucosal secretions were collected from the immunized mice 13-14 days after the last immunization, and the levels of OVA-specific antibodies were measured. As shown in Figure 2(a), i.n. immunization with TCTA1T enhanced the OVA-specific serum IgG1 and IgG2a titers to levels that were comparable to those in mice immunized with CT. TmCTA1T lacking ADP-ribosyltransferase activity also enhanced the magnitude of OVA-specific serum IgG response compared to the control group that received OVA alone, but the levels of OVA-specific IgG1 and IgG2a titers were considerably lower than those detected in mice immunized with TCTA1T. Meanwhile, the levels of OVA-specific serum IgG1 and IgG2a titers elicited by CTA1 were 4-fold lower than those induced by TCTA1T (data not shown). In addition, we observed that IgG1 was the predominant isotype in the sera of mice that had received OVA with TCTA1T, TmCTA1T, or CT (Figure 2(a)).

Various aspects of mucosal immunity serve critical functions in the defense against respiratory pathogens; one of which is the production and secretion of mucosal IgAs [3]. To examine whether adjuvantation of OVA with TCTA1T also enhances the mucosal IgA responses, OVA-specific mucosal IgA titers were examined in saliva, nasal wash, BAL, and lung tissues of the immunized mice. While i.n. delivery of OVA alone failed to induce OVA-specific IgAs, significant levels of OVA-specific sIgAs were observed in all external secretions of mice that received OVA immunization with TCTA1T or CT. Meanwhile, immunization with TmCTA1T elicited moderately higher levels of OVA-specific sIgAs in nasal wash, BAL, and lung tissues than those elicited by immunization with OVA alone (Figure 2(b)). Hence, these results indicate that adjuvantation of an Ag with TCTA1T enhances the Ag-specific mucosal sIgA secretion upon i.n. delivery and that ADP-ribosyltransferase activity may be associated with the immune-enhancing functionality of TCTA1T.

Further, in order to determine the optimal dose of TCTA1T to be used in mucosal immunizations, BALB/c mice were immunized i.n. three times with OVA mixed with 0.1, 1.0, 10, or 20 *μ*g of TCTA1T. The levels of OVA-specific IgG and IgA in sera and saliva were increased in a dose-dependent manner following immunization, and the observed dose-dependent increase in OVA-specific Ab titers peaked at 10 *μ*g dose for both serum IgG and salivary IgA (Figures 2(c) and 2(d)). Taken together, these results suggest that i.n. administration of TCTA1T as a mucosal adjuvant effectively enhances the systemic and mucosal Ab responses to the coadministered Ag.

### 3.4. TCTA1T Enhances *In Vivo* Ag-Specific Cytotoxic T Lymphocyte Response

**Figure 3:**
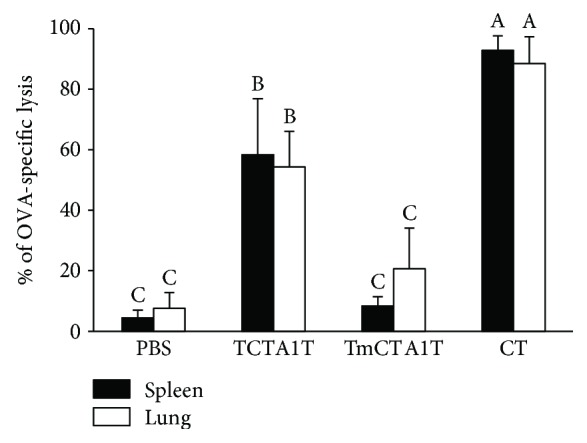
TCTA1T promotes OVA-specific CTL responses. C57BL/6 mice (*n* = 3 to 6) were immunized with 100 *μ*g of OVA alone, plus TCTA1T, TmCTA1T, or CT on days 0, 14, and 28. The immunized mice were injected i.v. with OVA peptide-pulsed and nonpulsed target cells. OVA-specific target cell lysis was determined in the spleen and lungs by flow cytometry. Data are expressed as mean ± SD. Data are representative of three separate experiments. The different letters within the same samples are significantly different (*P* < 0.05).

Cytotoxic T lymphocyte (CTL) response in the mucosal tissues plays a crucial role in the clearance of viruses that infect mucosal epithelia [2], and coadministration of CT with a soluble protein Ag has been known to enhance Ag-specific CTL response [35]. To assess the CTL activity induced by TCTA1T, we performed *in vivo* CTL assay in the spleen and lung against the OVA_257–264_ epitope (SIINFEKL) which is recognized by H-2Kb MHC class I molecules. The *in vivo* CTL activity elicited by the TCTA1T was slightly lower than that by the CT but still much higher when compared to that by the TmCTA1T and OVA alone in the lung and spleen (Figure 3). Overall, these results indicate that mucosal immunization in the presence of TCTA1T effectively potentiates the induction of systemic and mucosal CTL response that is specific to the coadministered soluble Ag.

### 3.5. TCTA1T Adjuvant Enhances Protection against Lethal Infection with Influenza Virus

**Figure 4:**
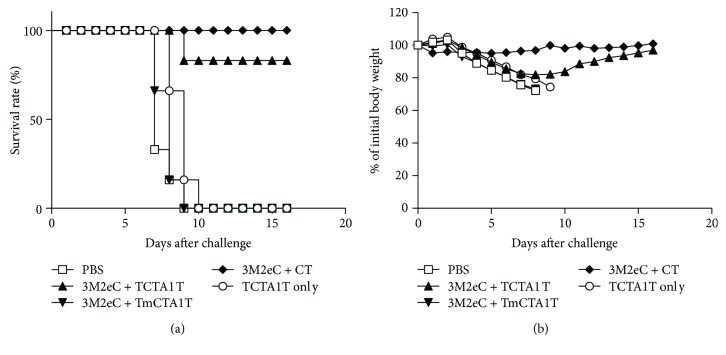
Protection against infection with A/PR/8 virus. BALB/c mice (*n* = 5) were immunized two times at 2-week intervals by i.n. administration with 10 *μ*g of influenza 3M2eC protein with 10 *μ*g of TCTA1T and TmCTA1T, or 2 *μ*g of CT as an adjuvant. Control groups were immunized with PBS or TCTA1T alone. Mice were challenged i.n. with 10 LD_50_ of A/PR/8 virus three weeks after the last immunization. Survival rate (a) and body weight (b) were monitored daily after the challenge. Data are representative of three separate experiments.

We have previously reported that mucosal vaccination of recombinant influenza virus M2 protein with CT offers better protection against lethal influenza virus challenge than parenteral vaccination [30]. In view of better protection offered by mucosal vaccination, we further explored the potential of using TCTA1T as the mucosal adjuvant for a M2 protein-based influenza vaccine. Coadministration of recombinant influenza M2 protein with TCTA1T or CT provided almost complete protection against lethal influenza virus challenge (survival rate 83.3% and 100%, resp.), whereas none of the mice immunized with M2 protein with TmCTA1T or control groups (PBS or TCTA1T alone) survived after challenge with 10 LD_50_ of influenza virus (Figure 4(a)). Furthermore, we observed a significant body weight loss in the mice immunized with M2 protein with TmCTA1T or control groups compared to mice that received M2 protein with TCTA1T or CT (Figure 4(b)). These results demonstrate that mucosal immunization of mice with influenza M2 protein in mixture with TCTA1T enhances the protective immunity against influenza virus challenge and provide evidential support for effectiveness of TCTA1T as a potential adjuvant for M2-based mucosal influenza vaccines.

### 3.6. TCTA1T Adjuvant Is Safe

**Figure 5:**
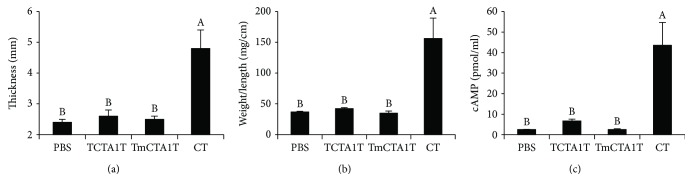
*In vitro* and *in vivo* toxicities of TCTA1T. (a) 20 *μ*g of TCTA1T and TmCTA1T or 1 *μ*g of CT in 10 *μ*l of PBS was injected into the hind paw. The thickness of the footpad was measured after 24 hrs. (b) Mice were anesthetized with ketamine, and abdomen was opened to make a loop in the small intestine. Then, 10 *μ*g of TCTA1T and TmCTA1T, or 2 *μ*g of CT, in 100 *μ*l of PBS were injected into the loop. The ratio of weight to length was determined after 6 hrs and the data expressed as milligrams per centimeter (mg/cm) of fluid accumulation. (c) BHK21 cells were seeded at 1 × 10^6^ cells/well in a 6-well plate. The cells were incubated with 10 *μ*g of TCTA1T and TmCTA1T, or 1 *μ*g of CT for 3 hrs. The concentration of cAMP in the supernatant was measured by ELISA according to the manufacturer's instructions. The results are expressed as mean ± SD for the group (*n* = 3 to 4). Data are representative of two separate experiments. The different letters within the same samples are significantly different (*P* < 0.05).

**Figure 6:**
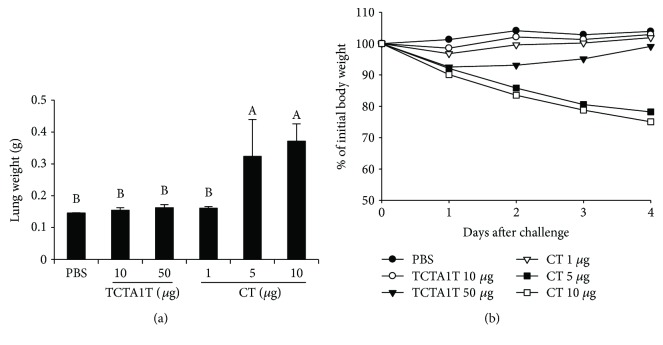
Comparison of toxicity effect between TCTA1T and CT in the lung tissue. The mice were administered i.n. with 10 or 50 *μ*g of TCTA1T or 1, 5, or 10 *μ*g of CT. PBS was used as a control. Lung tissues were removed from mice on day 4 after injection, and then (a) the weight of the lung was measured. (b) The mice were monitored daily for loss weight. The results are expressed as mean ± SD for the group (*n* = 5). The different letters within the same samples are significantly different (*P* < 0.05).

It is well known that CT possesses a potent adjuvant activity. However, it is not licensed for human use because of its toxicity [6]. To assess the potential toxicity of TCTA1T, three different CT toxicity tests—footpad edema, the intestinal loop, and the cAMP secretion test—were performed with minor modifications. Treatment of mice with CT proved toxic *in vivo* as shown by the results of footpad edema (Figure 5(a)) and intestinal loop tests (Figure 5(b)), whereas no evident sign of toxicity was observed following TCTA1T treatment. In addition, treatment of BHK21 cells with TCTA1T induced a marginal secretion of cAMP in levels significantly lower than the cAMP secretion caused by CT treatment (Figure 5(c)). The ADP-ribosylating activity produced by TCTA1T treatment was approximately 15% in value of that produced by CT treatment, which is comparable in ratio to the ADP-ribosylating activity exhibited by CTA1-DD treatment as reported in a previous study [18]. Next, we further examined the toxicity associated with i.n. administration of TCTA1T in the lung tissue of the mice. Administration with 5 *μ*g of CT resulted in a considerable increase in lung weight and caused significant body weight loss. However, no significant increase in the lung tissue weight or body weight loss was observed in the mice that received 10 or 50 *μ*g of soluble TCTA1T (Figures 6(a) and 6(b)). Taken together, these results underscore the lack of toxicity associated with TCTA1T in both *in vitro* and *in vivo* models advocating the safety of TCTA1T as a mucosal adjuvant.

### 3.7. TCTA1T Is Not Immunogenic

**Figure 7:**
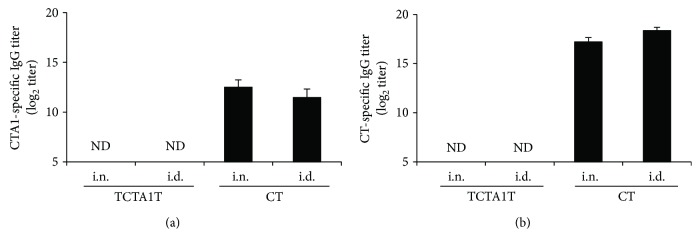
TCTA1T is not immunogenic. BALB/c mice were immunized three times with 2 *μ*g of CT or 10 *μ*g of TCTA1T by i.n. or i.d. routes. Sera were collected on day 14 after the last immunization. Levels of (a) CTA1- and (b) CT-specific IgG Abs in sera were determined by ELISA. The results are expressed as mean ± SD for the group (*n* = 3). ND stands for not detectable.

Since the presence of preexisting Abs to an adjuvant may inhibit immune response against codelivered Ags [23, 24], we evaluated the intrinsic immunogenicity of TCTA1T. Briefly, BALB/c mice were immunized three times i.n. or i.d. with 2 *μ*g of CT or 10 *μ*g of TCTA1T. Two weeks after the last immunization, the levels of CTA1- or CT-specific serum Ab were measured by ELISA. While CT also induced CT- or CTA1-specific serum IgGs following i.n. and i.d. administration, no detectable levels of CTA1- or CT-specific serum IgGs were observed following TCTA1T-adjuvanted immunization via either routes (Figures 7(a) and 7(b)). These results suggest that TCTA1T is not intrinsically immunogenic, even after repeated administration, and should thus be considered as an attractive adjuvant candidate for enhancing immune responses to coadministered vaccine antigens.

## 4. Discussion

In this study, we demonstrate that a novel recombinant fusion polypeptide (TCTA1T), derived from CT by replacing the CTB subunit with HIV-1 Tat PTD, has potent adjuvanticity, being capable of enhancing systemic and mucosal Ab as well as CTL responses to intranasally (i.n.) coadministered antigen. In addition, we demonstrate that i.n. delivery of influenza M2 protein with TCTA1T provides near complete protection against lethal influenza virus challenge. Furthermore, contrary to CT, TCTA1T lacked toxicity and self-immunogenicity, providing a rationale for the potential use of TCTA1T as a mucosal adjuvant due to its safety and ability to enhance both humoral and cellular immunities to the codelivered Ag.

CT is a well-known mucosal adjuvant in experimental animals but its toxicity precludes its use in humans [6]. In its native state, CTA1 subunit is internalized into the cell cytoplasm following binding of CTB subunits to cell surface GM1 ganglioside receptors, resulting in increased intracellular cAMP levels, disruption of ion channels, and hypersecretion of electrolytes and water leading to severe diarrhea [13, 14]. Our goal was to eliminate the toxicity of CT while retaining its adjuvanticity. To this end, we engineered a novel CTA1-based adjuvant capable of HIV-1 Tat protein PTD-dependent cellular entry, bypassing the need for the presence of CTB for the cellular translocation of CTA1. Our rationale for fusing HIV-1 Tat PTD to CTA1 emanated from the previous studies describing the ability of HIV-1 Tat PTD to permeate across the cell membrane in a receptor-independent manner [36, 37] and to deliver PTD fusion proteins into living cells [26, 27]. Accordingly, we have successfully shown in our study that using TCTA1T as mucosal adjuvant was effective in eliciting desired immune responses against the coadministered Ag. Also, given that TCTA1T lacks CTB domain, our finding indicates that CTB domain may be dispensable for the adjuvanticity of CT. This notion is also supported by the previous finding that CTA domain determines the adjuvanticity of CT [38].

However, the question of whether a direct correlation exists between the ADP-ribosyltransferase activity and the adjuvanticity of CT still remains controversial. Previous studies have demonstrated that two CT mutants, designated S61F and E112K, containing a point mutation within the ADP-ribosyltransferase active site of CTA1 domain retained their adjuvanticity without toxicity [39, 40], indicating that ADP-ribosyltransferase activity may not be required to establish the adjuvanticity of CT. Based on our study, however, while both TCTA1T and TmCTA1T were minimally or not toxic, TCTA1T, which possesses approximately 15% of the enzymatic activity of the intact CT, induced much higher mucosal and systemic Ab and CTL responses than those induced by TmCTA1T, in which ADP-ribosyltransferase functionality had been inactivated. In line with our findings, another study has reported that CTA1-DD possessing 10–20% of ADP-ribosyltransferase activity of the CT holotoxin, but, not mCTA1 E112K-DD, completely lacking the ADP-ribosyltransferase activity, induced significant immune responses to codelivered Ag following i.n. administration [18]. Taken together, our results support the notion that ADP-ribosyltransferase activity within CTA1 is essential for retaining the adjuvanticity of CT.

Importantly, we also demonstrate that TCTA1T does not exhibit *in vitro* or *in vivo* toxicity as shown through various toxicity tests, and the absence of toxicity following TCTA1T treatment may be associated with the cellular transport and location of TCTA1T which may differ from that of CT. Given the disparity in mechanism for cellular entry, it is possible to consider that TCTA1T and CT are transported to different sites within cells upon entry, and such differential cellular localization may contribute to the establishment of their toxicity. It is also possible that the addition of extra amino acids to the N-terminus of CTA1 via Tat PTD fusion may have caused steric interference on ADP-ribosyltransferase catalytic site and reduced toxicity of TCTA1T. This is in accordance with the observation made in a previous study that an increasing peptide chain length at the N-terminus of CTA is inversely correlated to its toxicity [41]. However, mechanism through which the absence of toxicity associated with TCTA1T observed in our present study is to be further investigated.

Another important requirement for an ideal adjuvant is the absence of immunogenicity to itself [42]. Preexisting immunity to an adjuvant could potentially interfere with adjuvant activity by preventing its uptake by antigen-presenting. Preexisting immunity to CTB has been shown to have a negative effect on the induction of immune responses to protein Ags conjugated to CTB [23, 24]. Unfortunately, some bacteria toxin-derived mutants were shown to induce significant antitoxin immunity, although adjuvant-reactive Abs did not appear to interfere with their adjuvanticity [21, 22]. Nonetheless, our data indicate that TCTA1T was poorly if at all immunogenic per se, minimizing the concerns of a potential loss of adjuvanticity arising from preexisting antiadjuvant immunity.

## 5. Conclusion

In conclusion, our study demonstrates that recombinant TCTA1T is a potent and effective candidate mucosal adjuvant, being capable of enhancing both humoral and cellular immune responses to a coadministered antigen. Being minimally toxic and if at all immunogenic, TCTA1T possesses attractive properties to be considered as a safe and efficient mucosal adjuvant.

## Abbreviations

Ab: Antibody
Ag: Antigen
BAL: Bronchoalveolar lavage
cAMP: Cyclic AMP
CT: Cholera toxin
CTA: Cholera toxin A subunit
CTB: Cholera toxin B subunit
CTL: Cytotoxic T lymphocyte
HIV-1: Human immunodeficiency virus type 1
i.d.: Intradermal
i.n.: Intranasal
i.p.: Intraperitoneal
i.v.: Intravenous
LT: Heat-labile toxin
GM1: Monosialoganglioside
PTD: Protein transduction domain
sIgA: Secretory IgA
Tat: Transcriptional activator
TCTA1T: Attached protein of PTD at both N-terminus and C-terminus of CTA1
TmCTA1T: Mutant TCTA1T
TLR: Toll-like receptor.
